# Age-Related Multigene Analysis of Colorectal Cancer Using Next-Generation Sequencing

**DOI:** 10.3390/cancers17243909

**Published:** 2025-12-06

**Authors:** Monika Kozlowska-Geller, Łukasz Nawacki, Monika Wawszczak-Kasza, Wojciech Lewitowicz, Jacek Bicki, Piotr Lewitowicz

**Affiliations:** 1 Collegium Medicum, Jan Kochanowski University in Kielce, 25-317 Kielce, Poland; 2 Department of Medical Genetics and Laboratory Diagnostics, Collegium Medicum, Jan Kochanowski University in Kielce, 25-317 Kielce, Poland; 3 Meduniv Sp.z o.o Al. IX Wiekow Kielc, 25-317 Kielce, Poland

**Keywords:** genomic era, next-generation sequencing (NGS), personalized medicine (PM), colorectal cancer (CRC), age-related molecular profiling, multigene panel analysis, genotype-phenotype correlation

## Abstract

Background: Colorectal cancer (CRC) remains a major global health problem, with rising incidence among younger individuals. The implementation of next-generation sequencing (NGS) has enabled comprehensive multigene analysis to identify cancer-predisposing variants and molecular alterations in tumors. However, data on age-related genetic differences in CRC from Central and Eastern European populations, including Poland, remain limited. Methods: This study aimed to explore molecular differences in CRC between patients aged ≤50 and >50 years in a Polish cohort. Tumor DNA was extracted from formalin-fixed, paraffin-embedded (FFPE) tissue samples obtained from 54 treatment-naive patients. Targeted sequencing of hot spot regions of 50 genes with known association to cancer was performed using an AmpliSeq for Illumina Cancer Hotspot Panel v2. Results: Variant frequencies in younger vs. older patients were: *TP53* (71.4% vs. 57.6%), *APC* (57.1% vs. 45.5%), *KRAS* (28.1% vs. 72.7%), *NRAS* (28.6% vs. 0%), *SMAD4* (9.5% vs. 12.1%), *PIK3CA* (14.3% vs. 24.2%), and *FBXW7* (4.8% vs. 14.7%). Co-occurrence of *APC*/*KRAS*/*TP53* variants was observed in 20% of cases. *KRAS* mutations were significantly more frequent in older patients (*p*-value = 0.001), while *NRAS* mutations occurred exclusively in younger patients (29% vs. 0%, *p* = 0.021). Overall, 46% of patients exhibited multiple gene alterations (≥3 mutations). Notably, *IDH1* and *CTNNB1* variants were found only in patients with better prognosis, whereas *TP53* variants were nearly five times more frequent in patients with worse outcomes. Conclusions: Multigene panel sequencing revealed distinct age-related molecular patterns in CRC. Younger patients were more likely to harbor *NRAS* variants, whereas *KRAS* alterations predominated in older individuals. These findings underscore the relevance of NGS-based multigene profiling for risk stratification and personalized therapy in colorectal cancer.

## Simple Summary

Colorectal cancer is one of the leading causes of cancer-related death worldwide, and its incidence among younger individuals is steadily increasing. Early identification of patients at higher risk of poor outcomes is therefore crucial. In this study, we analyzed molecular differences between younger and older colorectal cancer patients from a Polish cohort using next-generation sequencing (NGS). This work provides valuable data from the Polish population, where such genomic studies remain limited, and helps to better understand how age-related molecular profiles may influence prognosis and personalized treatment strategies in colorectal cancer.

## 1. Introduction

Colorectal cancer (CRC) is the third most diagnosed malignancy and the second leading cause of cancer-related mortality worldwide, accounting for nearly 2 million new cases and 1 million deaths annually [1]. Early-onset colorectal cancer (EOCRC) is defined as colorectal cancer diagnosed in individuals younger than 50 years of age. This definition is widely used in medical literature and aligns with the age threshold at which most population-based colorectal cancer screening programs begin for average-risk individuals [2,3,4,5,6].

The increasing incidence of colorectal cancer (CRC) in younger adults was first documented in analyses of data from the Surveillance, Epidemiology, and End Results (SEER) program, covering the late 20th century [7]. While CRC rates in older adults remained stable or declined, a steady rise was observed in individuals under 50 years of age, who were also more frequently diagnosed at advanced stages [7]. Over the past three decades, this pattern has persisted, with incidence among adults aged 20–49 increasing by nearly 45% and mortality in this age group rising annually, in contrast to the continuing decline observed in older populations [8,9]. The increase, consistent across both sexes, varies by tumor site (predominantly rectal and distal colon), disease stage, race, ethnicity, and geographic region [8]. Most cases of early-onset colorectal cancer (EOCRC) are sporadic; however, a markedly higher proportion of patients in this age group present with hereditary cancer syndromes—most notably Lynch syndrome—compared with individuals diagnosed at a later age [2,3,4]. EOCRC is also more frequently diagnosed at an advanced stage and is characterized by a predominance of tumors arising in the distal colon or rectum [4,9,10]. Given these clinicopathological distinctions, growing attention has been directed toward identifying the underlying risk factors that predispose younger individuals to the development of EOCRC.

Risk factors include both non-modifiable factors (family history, hereditary syndromes, inflammatory bowel disease) and modifiable factors (obesity, poor diet, sedentary lifestyle, smoking, alcohol) [3]. The American Cancer Society recommends starting colorectal cancer screening at age 45 for average-risk individuals to address the rising incidence of early-onset disease [2]. Case–control studies have identified several potential risk factors for early-onset colorectal cancer (CRC), including male sex, race (particularly Black and Asian ethnicities), a family history of CRC, alcohol consumption, weight loss of ≥5 kg within five years prior to colonoscopy, processed meat intake, and inflammatory bowel disease (IBD). In contrast, regular aspirin use and higher consumption of vegetables, citrus fruits, fish, β-carotene, vitamin C, vitamin E, and folate have been linked to a reduced risk of early-onset CRC [4,10].

Early-onset CRC most frequently arises in the rectum (35–37%), followed by the distal colon (25–26%) and the proximal colon (22–23%). In comparison, among individuals aged >50 years (later-onset CRC), approximately 29% of cases occur in the rectum, 27% in the distal colon, and 29% in the proximal colon [6].

Patients with early-onset CRC are more likely to present with advanced disease, poor tumor differentiation, and aggressive clinical features, indicating potential biological differences compared with late-onset CRC [4,11]. The mutational patterns observed in EOCRC differ from those in later-onset disease in several key respects. EOCRC demonstrates a higher prevalence of pathogenic germline variants, particularly in DNA mismatch repair (MMR) genes (*MLH1*, *MSH2*, *MSH6*, *PMS2*), which are characteristic of Lynch syndrome and result in microsatellite instability-high (MSI-H) tumors. Approximately 16–25% of EOCRC cases harbor germline variants, with about half attributable to Lynch syndrome [4,6,10,11,12,13].

In sporadic, microsatellite-stable (MSS) EOCRC, somatic variant profiles show increased frequency of *TP53* and *CTNNB1* variants, and decreased frequency of *APC*, *KRAS*, and *BRAF* variants compared to later-onset CRC. Notably, *BRAF* V600E and CpG island methylator phenotypes (CIMP-high) are less common in EOCRC, especially in rectal and left-sided tumors. Changes in *KRAS* codon 12 and enrichment of *NOTCH1*, *FBXW7*, *PIK3CA*, and *FGFR3* variants are more frequent in EOCRC than in adult-onset cases [14,15,16,17].

Hypermutated EOCRC tumors exhibit higher tumor mutational burden (TMB) and a higher prevalence of somatic variants in *APC*, *KRAS*, *CTNNB1*, and *TCF7L2*, while *BRAF* and *RNF43* variants are less frequent compared to late-onset hypermutated CRC [3]. Somatic variants profile in EOCRC MSS tumors include *FBXW7* with *NOTCH3*, *RB1*, and *PIK3R1*.

In summary, EOCRC is characterized by a higher rate of germline MMR variants, increased *TP53* and *CTNNB1* alterations, lower incidence of *APC*, *KRAS*, and *BRAF* variants, and unique co-mutational patterns, supporting the need for age-specific molecular profiling.

While these mechanisms are well established, accumulating evidence suggests that the distribution and frequency of driver variants may vary by age. Understanding these age-related genomic differences is critical for risk stratification, early detection, and individualized therapeutic strategies [17,18,19,20,21].

Next-generation sequencing (NGS) has transformed cancer genomics by enabling high-throughput, multigene molecular profiling with substantially enhanced sensitivity, analytical depth, and cost-efficiency. In contrast to conventional sequencing methods, NGS facilitates comprehensive detection of single-nucleotide variants, insertions and deletions, copy-number alterations, and gene fusions across extensive cancer-associated gene panels. Recent advances in NGS technologies and their pivotal role in uncovering previously unrecognized genomic features of colorectal cancer have significantly advanced our understanding of CRC tumorigenesis and expanded opportunities for identifying clinically actionable targets in the context of precision oncology. In CRC, NGS is routinely employed to detect therapeutically and prognostically relevant alterations in genes including *KRAS*, *NRAS*, *BRAF*, *PIK3CA*, *TP53*, and the mismatch-repair (MMR) genes [22]. However, systematic age-stratified multigene analyses of CRC using NGS remain limited.

This study aims to characterize age-related genomic alterations in colorectal cancer through multigene analysis using next-generation sequencing. By comparing mutational landscapes between younger and older patients, we seek to elucidate potential biological differences that may underlie variations in clinical presentation and outcomes. Such insights may contribute to the refinement of age-specific screening strategies, prognostic models, and precision therapeutic approaches.

## 2. Materials and Methods

This retrospective analysis included data from colorectal cancer (CRC) molecular diagnostics performed between 2022–2024 based on tumor samples obtained from Department of Clinical and Experimental Pathology Meduniv in Kielce, Poland. All clinical data were anonymized prior to analysis. Patients with histopathologically confirmed colorectal adenocarcinoma, not otherwise specified (NOS), were included. Moreover, to keep a homogeneous cohort of adenocarcinoma NOS, all immunohistochemically confirmed microsatellite-instable tumors were rejected. Additional exclusion criteria included prior radiotherapy or chemotherapy. The final study cohort comprised 54 patients, including 29 men (53.7%) and 25 women (46.3%). The mean age at diagnosis was 55 years (range: 31–92 years). All cases were re-diagnosed according to UICC TNM-8ed.

### 2.1. DNA Isolation

Genomic DNA was extracted from formalin-fixed paraffin-embedded (FFPE) tumor tissue. The isolation was performed from tissue fragments obtained by scalpel microdissection, following prior delineation of tumor-rich regions by a pathologist based on hematoxylin and eosin (H&E)-stained slides. The proportion of tumor cells within the dissected areas ranged from 60% to 100%.

Extraction was performed using the MagCore Automated Extraction Kit No. 405 (MagCore, RBC Bioscience, New Taipei, Taiwan), following the manufacturer’s protocol. DNA concentration and purity were assessed using the Quantus^®^ Fluorometer (Promega, Madison, WI, USA) and the QuantiFluo ONE dsDNA Kit (Promega, Madison, WI, USA).

### 2.2. Library Preparation and Sequencing

Amplicon-based analysis was performed to target hotspot regions in 50 oncogenes and tumor suppressor genes. Libraries were prepared with the AmpliSeq Library PLUS for Illumina^®^ kit (San Diego, CA, USA) according to the AmpliSeq for Illumina Cancer HotSpot Panel v2 Reference Guide. This panel generates 207 gene-specific amplicons and covers approximately 2800 clinically relevant mutations.

Amplification: Multiplex PCR (HotSpot Panel v2) was performed using DNA diluted to a final concentration of ~30 ng/reaction.

Adapter ligation: Adapters from the AmpliSeq CD Indexes Set A were ligated to the amplified DNA.

Purification: Amplicons were purified using NucleoMag^®^ NGS Clean-up and Size Select beads (Macherey-Nagel GmbH & Co., Düren, Germany).

Quantification and pooling: Libraries were quantified with the QuantiFluo^®^ ONE dsDNA System (Promega, Madison, WI, USA), normalized to 4 nM, and equimolarly pooled.

Size analysis: Fragment distribution was assessed with the 4150 TapeStation System (Agilent Technologies, Santa Clara, CA, USA).

Sequencing was carried out on the Illumina MiSeq Dx(San Diego, CA, USA) platform using the MiSeq Reagent Micro Kit v2 (300 cycles). Libraries were denatured with NaOH and diluted to a final concentration of 20 pM. A 9 pM working library was prepared with a 5% spike-in of PhiX Control v2 (Illumina, San Diego, CA, USA) to monitor sequencing quality.

### 2.3. Genomic Data Analysis

Analysis of NGS data was performed using the GALAXY platform (usegalaxy.org accessed on 10 February 2024). Sequencing reads (FASTQ files) were aligned to the human reference genome hg19 using the Bowtie2 tool. Variant calling was performed using the Varscan2 tool. The parameters used for data analysis were minimum allele frequency, 0.05; minimum quality, 20; and minimum coverage ×80. All called variants were annotated using WANNOVAR (https://wannovar.wglab.org (accessed on 10 February 2024)). Results were visualized using the R Bioconductor package Maftools (http://bioconductor.org/ (accessed on 10 February 2024)). The pathogenicity of individual variants was determined using the Franklin (Genoox) evidence-based classification system, ensuring standardized and clinically relevant interpretation of detected alterations. Synonymous variants were not included in the analysis, as only non-synonymous alterations with potential functional relevance were considered.

### 2.4. Statistical Analysis

Clinicopathological and molecular data were analyzed using SPSS Statistics v22 (IBM Corp., Armonk, NY, USA). Continuous variables are presented as mean ± SD and range; categorical variables are expressed as percentages. Group comparisons were performed using:

χ^2^ test or Fisher’s exact test for categorical variables;

Mann–Whitney U test or Student’s t-test for continuous variables;

A two-sided *p*-value ≤ 0.05 was considered statistically significant.

Comparisons of mutation frequencies between early-onset (EOCRC, ≤50 years) and later-onset (LOCRC, >50 years) colorectal cancer were performed using contingency tables and appropriate tests for categorical variables. Mutation status for each gene was coded as binary (mutated vs. wild type). For each gene, we constructed a 2×2 table contrasting EOCRC vs. LOCRC and calculated odds ratios (OR) with 95% confidence intervals (CI). When any cell count was <5, Fisher’s exact test was used; otherwise, the chi-square test without continuity correction was applied.

Because multiple genes were evaluated in parallel, raw *p*-values were adjusted for multiple comparisons across genes using the Benjamini–Hochberg false discovery rate (FDR). As a sensitivity analysis, Bonferroni-corrected *p*-values were also computed. In the presence of zero counts, odds ratios and CIs were estimated using the Haldane–Anscombe correction (adding 0.5 to each cell). A two-sided FDR-adjusted *p*-value <0.05 was considered statistically significant.

## 3. Results

### 3.1. Clinical Features

Patients were divided into two age groups: ≤50 years (*n* = 21) and >50 years (*n* = 33). Clinicopathological features are summarized in Table 1.

The median age was 43 years in the younger group and 63 years in the older group. The female-to-male ratio was 1.2:1. No significant differences were observed in mean age or sex distribution between groups.

### 3.2. Somatic Mutations in the Whole Cohort

**Figure 1:**
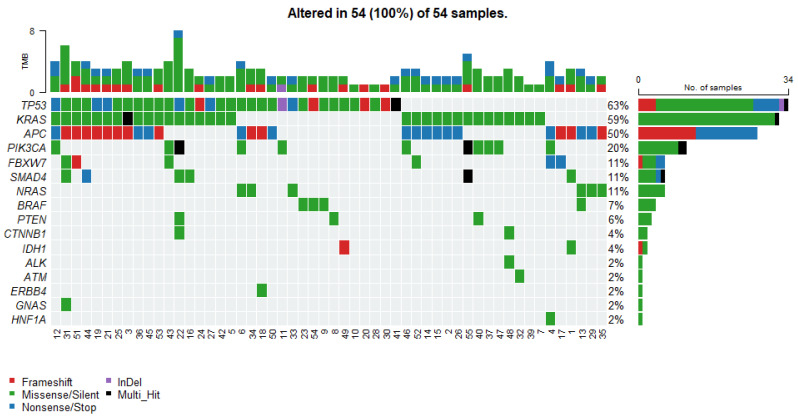
Oncoplot displays the somatic mutation distribution of the top highly mutated genes. Each column represents a tumor, and the bar graph (tumor mutation burden—TMB) at the top shows the number/distribution of mutations detected per sample. The Oncoprint rows show the changes for each gene. The bar graph on the right side of the panel shows the number and distribution of mutations for each gene. Mutation types are color-coded according to the legend.

The overall mutation landscape is illustrated in Figure 1 and summarized in Table 2.

The most recurrent alterations were observed in *TP53*, mutated in 34 cases (63%), followed closely by *KRAS* mutations detected in 32 cases (59%). APC alterations were identified in 27 tumors (50%). Less frequent but still notable mutations included *PIK3CA* in 11 cases (20%). Variants in *NRAS*, *SMAD4* and *FBXW7* each occurred in 6 patients (11%). Mutations in *BRAF* and *PTEN* were relatively rare, identified in 4 (7%) and 3 (6%) cases, respectively. The frequently occurring somatic variants, together with the corresponding clinical data, are presented in Supplementary Table S1.

Table 2 summarizes all rare somatic variants observed in the study cohort, including the affected genes, associated protein, variant types, and their clinical significance according to the Franklin database.

Tumor mutational burden, defined in this study as the total number of detected somatic variants per case, ranged from 1 to 8. The distribution was as follows: TMB = 2, being the most common category (23 cases, 42.6%), followed by TMB = 3 (15 cases, 27.8%) and TMB = 1 (6 cases, 11.1%). Higher TMB values occurred infrequently, including TMB = 4 (7 cases, 13.0%), TMB = 5 (1 case, 1.9%), TMB = 6 (1 case, 1.9%), and TMB = 8 (1 case, 1.9%). No tumors exhibited TMB = 7 (Figure 1). The overall distribution indicated that the cohort was predominantly characterized by low TMB values, with a median of 2 variants per case.

**Figure 2:**
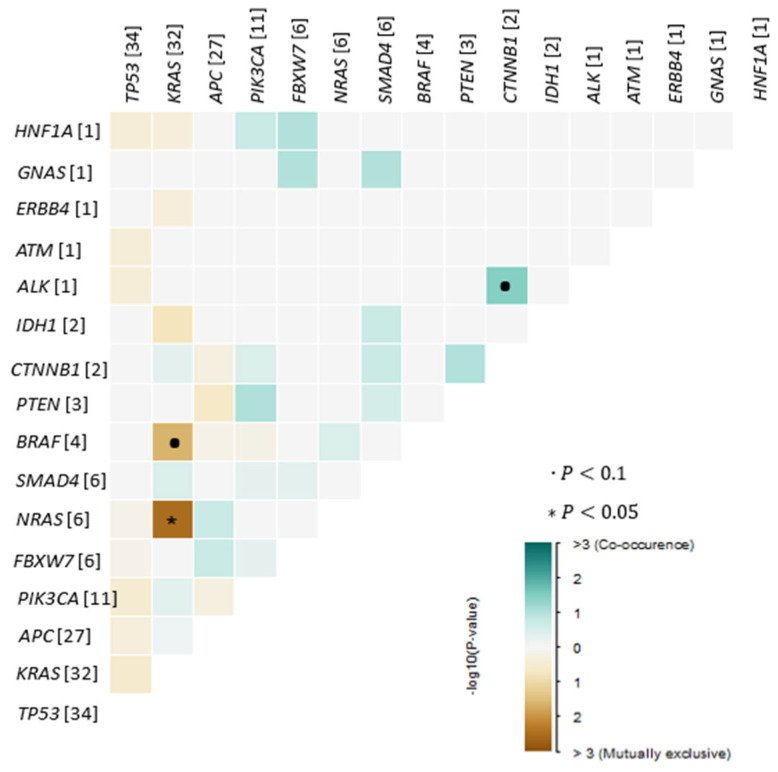
Exclusive/co-occurrence event analysis of the top 25 mutated genes (*p* < 0.05, pairwise Fisher’s Exact test).

Analysis of co-occurrence patterns revealed that most tumors harbored more than one somatic alteration, with frequent combinations involving canonical CRC driver genes. We detected the following statistically significant correlations (*p* < 0.05, pairwise Fisher’s Exact test): a negative association between NRAS and KRAS mutations, reflecting their mutual exclusivity within the cohort (Figure 2). The most common co-mutational pattern included simultaneous alterations in *APC*, *TP53*, and *KRAS*, reflecting the classical adenoma–carcinoma sequence and representing the dominant molecular backbone across the cohort. Additional recurrent co-occurring events included *TP53*–*PIK3CA*, *KRAS*–*PIK3CA*, and *TP53*–*SMAD4* combinations, each observed in multiple cases.

Several tumors exhibited more complex mutational profiles, with four or more variants detected within the same sample, indicating greater genomic instability even within microsatellite-stable (MSS) disease. Co-occurrence patterns also varied across rare variants: for example, *CTNNB1*, *IDH1*, and *GNAS* mutations were found exclusively in tumors that already carried alterations in major driver genes such as *APC*, *TP53*, or *KRAS*, suggesting that these rare events function as secondary or modifying mutations rather than primary drivers.

### 3.3. Somatic Variant Distribution Across EOCRC and Non-EOCRC Groups

Age-stratified analysis demonstrated distinct differences in the distribution of somatic variants between EOCRC and older patients (Table 3).

Pairwise Fisher’s Exact tests revealed two statistically significant age-related differences in the mutational landscape. However, the significance threshold for the Bonferroni correction is α/m = 0.05/8 = 0.00625. At this threshold, only *NRAS* (*p* raw = 0.0021) remains statistically significant after correction (*p* Bonferroni = 0.0168), whereas *KRAS* shows a strong trend (*p* Bonferroni = 0.093). No other genes demonstrated significant age-associated differences, including *TP53*, *APC*, *PIK3CA*, *SMAD4*, *FBXW7*, or *BRAF*. Rarely altered genes such as *IDH1*, *GNAS*, *ATM*, *ALK*, *ERBB4*, *HNF1A*, and *CTNNB1* also showed no statistically significant differences between groups. These findings indicate that while the overall driver profile is broadly similar across age categories, RAS-pathway alterations—specifically *KRAS* enrichment in older patients and *NRAS* restriction to EOCRC—constitute the primary statistically significant age-dependent differences in this cohort.

### 3.4. Variants and Prognosis

Somatic variants stratified by prognosis are summarized in Table 4. Staging was not determined for one patient.

*TP53* variants were nearly five times more frequent in patients with worse prognosis (28 vs. 6). *IDH1*, *CTNNB1*, *HNF1A*, and *ALK* variants occurred only in patients with favorable outcomes. *ERBB4*, *GNAS*, and *ATM* alterations were observed exclusively in those with poor prognosis.

## 4. Discussion

Clinical outcomes associated with early-onset colorectal cancer are characterized by a higher likelihood of advanced stage (III/IV) at diagnosis, more aggressive histopathologic features (including signet-ring cell and mucinous adenocarcinoma, and poor differentiation), and a greater frequency of recurrence, particularly in stage I disease. Despite these adverse features, crude overall survival and cancer-specific survival are generally similar to, or marginally better than, those seen in later-onset CRC after adjustment for stage and other prognostic factors [2,23,24,25,26].

Younger patients with EOCRC are more likely to receive intensive multimodality therapy, including higher rates of adjuvant and multiagent chemotherapy, but this does not translate into a substantial improvement in adjusted survival outcomes. In metastatic disease, EOCRC patients have similar overall survival compared to older patients, despite more aggressive treatment regimens. Long-term excess mortality remains elevated for both EOCRC and late-onset CRC, even beyond five years post-diagnosis, underscoring the need for extended survivorship care [26].

In a large study of 648 patients, Wang et al. [27] reported mutation frequencies of *TP53* (52.8%), *KRAS* (46.7%), *APC* (24.1%), *PIK3CA* (18.9%), *SMAD4* (9.5%), *BRAF* (6.1%), *FBXW7* (5.3%), and *NRAS* (4.1%). Our results are in close agreement, with *TP53* (63%), *KRAS* (59%), *APC* (50%), *PIK3CA* (20%), *SMAD4* (11%), *FBXW7* (11%), *NRAS* (11%), and *BRAF* (7%), confirming cross-population consistency in CRC mutational patterns. Although the overall incidence of CRC is decreasing, the burden among young adults has grown significantly in recent decades [27]. Risk factors such as obesity, alcohol, smoking, sedentary lifestyle, and diets high in red meat may contribute disproportionately to this rise in younger populations [28,29].

These results support findings by Chang et al. in Taiwan [30](*n* =1475), who also concluded that molecular differences between young and old CRC patients are limited, except for *KRAS* and *NRAS*, which showed significant age-dependent differences. Specifically, *KRAS* mutations were significantly more frequent in older patients (*p*-value = 0.001), whereas *NRAS* mutations were restricted to younger patients (*p*-value = 0.021).

*TP53* and *KRAS* mutations in early-onset colorectal cancer (EOCRC) are significant because they reflect distinct molecular features and have prognostic implications. In EOCRC, *TP53* mutations appear relatively frequent, particularly in microsatellite-stable tumors, suggesting a greater role for TP53-driven tumorigenesis in younger patients. *KRAS* mutations, while common in colorectal cancer overall, remain prevalent also in EOCRC, though their distribution may differ across cohorts [13,14,15].

Functionally, *TP53* mutations are associated with loss of tumor suppressor activity, contributing to genomic instability and aggressive tumor behavior. *KRAS* mutations, particularly in codons 12 and 13, activate downstream signaling pathways (MAPK, PI3K), promoting cell proliferation and resistance to anti-EGFR therapies [15]. The co-occurrence of *TP53* and *KRAS* mutations is associated with worse prognosis, including higher risk of recurrence and decreased survival, especially in metastatic settings. Specific combinations, such as *KRAS* codon 13 and *TP53* L3 domain mutations, further stratify risk for poor outcomes [16].

In summary, *TP53* alterations contribute to aggressive biology observed in EOCRC, while *KRAS* mutations remain clinically relevant due to their role in oncogenic signaling and treatment resistance.

The prevalence of multiple gene alterations (co-alterations) is higher in younger patients, with early-onset CRC showing more frequent co-occurrence of mutations such as *KRAS* with *ATM*, *ARID1A*, *CREBBP*, *FAT1*, *KMT2B*, and *KMT2D*, whereas these gene pairs are more often exclusive in late-onset CRC [16]. This pattern suggests that EOCRC may exhibit broader molecular complexity reflected by co-occurring mutations across several pathways.

Regarding prognosis, *TP53* mutations are associated with more aggressive tumor biology and worse clinical outcomes. *IDH1*, *CTNNB1*, *HNF1A*, and *ALK* mutations are also linked to poor prognosis in CRC, with patients harboring these mutations experiencing shorter progression-free and overall survival. The presence of multiple co-mutations, such as *TP53*/*IDH1* or *TP53*/*ALK*, further correlates with adverse outcomes. Other studies echo these observations. Chatsirisupachai et al. demonstrated that *TP53* and *CTNNB1* mutations were more common in younger CRC patients, while *APC*, *KRAS*, and *BRAF* V600E mutations predominated in older ones [31]. Our study supports these observations, showing a similar distribution of key driver mutations across age groups.

Most CRC cases are diagnosed in patients >50 years, but early-onset cases are increasing, now representing a substantial public health concern. Early detection and personalized treatment approaches are critical for this population [13,14,15]. Large retrospective studies, including one analyzing ~36,000 patients, have shown that early-onset CRC is more often associated with microsatellite instability (MSI), CTNNB1 and ATM mutations, and CIMP hypermethylation [30].

Our results also align with reports by Kim et al. [32], identifying recurrent alterations in known cancer-related genes (*APC*, *TP53*, *KRAS*, *PIK3CA*, *FBXW7*, *SMAD4*, *NRAS*). Prognostic implications are notable: *TP53* mutations were nearly five times more frequent in patients with worse prognosis, while *IDH1*, *CTNNB1*, *HNF1A*, and *ALK* mutations occurred only in patients with better outcomes [33,34,35]. These findings agree with Lipsyc, Kalady, and Shen, who linked *KRAS*, *NRAS*, *BRAF*, and *PIK3CA* mutations with poor survival [36,37,38]. *NRAS* has been implicated in inflammation-driven tumorigenesis, which may explain why we observe *NRAS* mutations only in younger patients.

Interestingly, *IDH1* mutations have been associated with younger age, favorable prognosis, and better therapeutic response [39,40]. Consistently, in our cohort, *IDH1* mutations appeared exclusively in patients with better prognosis.

Taken together, our results emphasize the clinical value of targeted NGS panels in CRC. Technology not only provides a comprehensive overview of recurrent mutations but also identifies rare genetic alterations that may carry prognostic or therapeutic implications. Future challenges lie in integrating NGS data into clinical workflows to refine patient stratification, improve early detection, and enable more precise treatment selection.

Extensive research is focused on developing synthetic modulators of Wnt signaling, including small molecules, peptides, and inhibitory antibodies that suppress this pathway. Lithium chloride, approved by the U.S. Food and Drug Administration (FDA), is already used clinically and is known to activate CTNNB1 by inhibiting GSK3. Additionally, nonsteroidal anti-inflammatory drugs (NSAIDs) and the selective COX-2 inhibitor celecoxib have been shown to block CTNNB1-dependent transcription in CRC [41,42,43,44].

Therapeutic strategies targeting the EGFR pathway typically utilize anti-EGFR monoclonal antibodies and tyrosine kinase inhibitors (TKIs) directed against intracellular kinases. Cetuximab was the first monoclonal antibody developed to target EGFR; upon binding to its extracellular domain, it induces receptor internalization and degradation. Multiple studies have demonstrated the positive impact of cetuximab on clinical outcomes in CRC patients [45,46].

Amivantamab is an EGFR-MET bispecific antibody with immune cell-directing activity and is FDA-approved for 4 indications in EGFR-mutated advanced non-small cell lung cancer. Recently, interesting data concerning advanced CRC has been published. In the phase 1b/2 OrigAMI-1 study (NCT05379595), amivantamab plus FOLFIRI demonstrated promising antitumor activity in patients with mCRC without prior anti-EGFR exposure [47].

## 5. Study Limitations

This study has several limitations. First, the relatively small cohort size, particularly after stratification by age group and clinical stage, reduces statistical power and may limit the detection of less frequent molecular differences between EOCRC and later-onset CRC. Second, the analysis was based on a single-center, retrospective dataset, which may introduce selection bias and restrict the generalizability of the results to wider populations, highlighting the need for validation in larger, internationally curated datasets such as TCGA, GEO, or other publicly available CRC cohorts.

Third, the molecular characterization relied on a targeted NGS panel. Although clinically relevant, this approach captures only a subset of genomic alterations, and panel-derived TMB values are not directly comparable with whole exome-based metrics. Consequently, structural variants, copy-number changes, and epigenetic alterations were not assessed. The interpretation of rare variants was also constrained by their very low frequency and dependence on silico classification tools such as Franklin, which do not always reflect biological function.

Finally, clinical outcome data were unavailable, precluding survival or treatment-response analyses. Because the EOCRC subgroup included a higher proportion of advanced-stage tumors, some observed differences may be influenced by stage distribution rather than intrinsic biological divergence. These factors should be taken into consideration when interpreting the findings.

## 6. Conclusions

**Figure 3:**
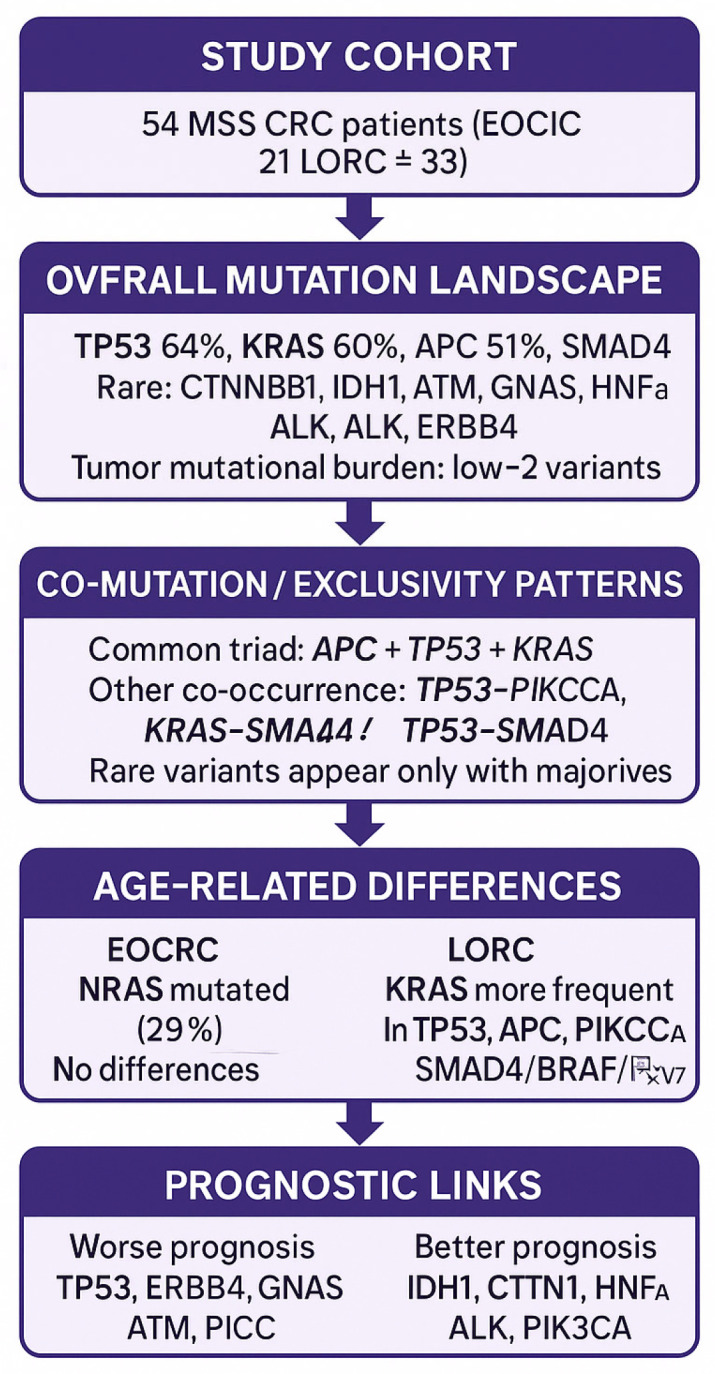
Graphical summary of results.

Molecular and clinicopathological differences between younger and older CRC patients were limited (Figure 3). However, *KRAS* mutations were significantly more frequent in older patients, and *NRAS* mutations were exclusive to younger patients.

In younger patients, the most common mutation was *TP53* (71.4%), while in older patients, *KRAS* (72.7%) predominated.

Almost half of patients (46%) harbored multiple gene alterations, most often three to five mutations.

*TP53* mutations were almost five times more frequent in patients with worse prognosis, while *IDH1*, *CTNNB1*, *HNF1A*, and *ALK* mutations were confined to patients with better prognosis.

The exclusive presence of *NRAS* mutations in young patients (29%) suggests an age-specific molecular pattern.

This targeted NGS assay proved suitable for clinical application, with potential relevance for diagnosis and prognosis in Polish CRC patients.

## Figures and Tables

**Table 1:** Clinicopathological features of the study cohort. EOCRC—early-onset colorectal cancer (≤50 years); LOCRC—later-onset colorectal cancer (>50 years).

Variable	All Patients (*n* = 54)	EOCRC (*n* = 21)	LOCRC (*n* = 33)
Median age (range)	55 (31–92)	43 (31–50)	63 (51–92)
Sex			
Male	29 (54%)	10	19
Female	25 (46%)	11	14
Stage	I: 9; II: 14; III: 17; IVA: 6; IVB: 8	I: 1; II: 3; III: 10; IVA: 6; IVB:1	I: 3; II: 5; III: 17; IVA: 5; IVB: 4

**Table 2:** Rare Somatic Variants Identified in the Study Cohort, Including Protein, Variant Types, and Franklin Pathogenicity Classification.

Gene	Protein Change	Variant Type	Franklin Classification
*CTNNB1*	p.S38F	missense	Pathogenic
*CTNNB1*	p.T34A	missense	Pathogenic
*IDH1*	p.R132H	missense	Pathogenic
*IDH1*	p.N116Kfs*27	frameshift	Likely pathogenic
*ALK*	p.R1181H	missense	VUS
*ATM*	p.R3008C	missense	Likely pathogenic
*ERBB4*	p.R938H	missense	VUS
*GNAS*	p.R201H	missense	Pathogenic
*HNF1A*	p.R200W	missense	Likely pathogenic

**Table 3:** Summary of Somatic Variants in EOCRC and LOCRC With Corresponding Franklin Pathogenicity Classification. EOCRC—early-onset colorectal cancer (≤50 years); LOCRC—later-onset colorectal cancer (>50 years); LP—likely pathogenic; VUS—variant of uncertain significance; NA—not available (variant not classified in Franklin); CRC—colorectal cancer; * *p*-value < 0.05.

Gene	EOCRC (*n* = 21)	LOCRC (*n* = 33)	Franklin Classification	*p*-Value Raw	*p*-Value (*Bonferroni*)
*KRAS*	8 (38.1%)	24 (72.7%)	Pathogenic / Likely pathogenic (G12/G13/A146 variants)	0.0116 *	0.093
*TP53*	15 (71.4%)	19 (57.6%)	Pathogenic / Likely pathogenic (hotspots, truncations)	0.304	1.0
*APC*	12 (57.1%)	15 (45.5%)	Somatic truncating APC variants not classified in Franklin	0.402	1.0
*PIK3CA*	3 (14.3%)	8 (24.2%)	Pathogenic (E542/E545/H1047), LP/VUS (others)	0.497	1.0
*NRAS*	6 (28.6%)	0 (0%)	Pathogenic (G12/G13/Q61 variants)	0.0021 *	0.0168 *
*BRAF*	2 (9.5%)	2 (6.1%)	Pathogenic (V600E), Likely pathogenic (G466R)	0.638	1.0
*SMAD4*	2 (9.5%)	4 (12.1%)	Pathogenic / VUS	1.0	1.0
*FBXW7*	1 (4.8%)	5 (15.2%)	Pathogenic (R465/R278), VUS (S582L)	0.386	1.0
*PTEN*	1 (4.8%)	1 (3.0%)	LP / VUS	NA	NA

**Table 4:** Variant distribution in patients with better vs. worse prognosis according to stage.

Gene (Total Cases in Cohort)	Stage I–II (Better Prognosis) N (%)	Stage III–IVB (Worse Prognosis) N (%)
*TP53* (*n* = 34)	6 (17.6%)	28 (82.4%)
*KRAS* (*n* = 32)	8 (25%)	24 (75%)
*APC* (*n* = 27)	9 (33.3%)	18 (66.7%)
*PIK3CA* (*n* = 11)	2 (18.2%)	9 (81.8%)
*NRAS* (*n* = 6)	2 (33.3%)	4 (66.7%)
*FBXW7* (*n* = 6)	1 (16.7%)	1 (83.3%)
Other rare variants (*n* = 9) *IDH1* (2), *CTNNB1* (2), *HNF1A* (1), *ALK* (1), *ERBB4* (1), *GNAS* (1), *ATM* (1)	4 (44.4%)	5 (55.6%)

## Data Availability

Research data supporting this publication are available upon request from Monika Kozłowska-Geller (monika.kozlowska.chir@onet.pl).

## Supplementary Materials

The following supporting information can be downloaded at: https://www.mdpi.com/article/10.3390/cancers17243909/s1, Table S1: Pathway Classification with Protein Changes.

## Abbreviations

*ALK*	anaplastic lymphoma kinase
*APC*	adenomatous polyposis coli
*ATM*	ataxia telangiectasia mutated
*BRAF*	B-Raf proto-oncogene, serine/threonine kinase
CD	cluster of differentiation (index set designation in AmpliSeq CD Indexes)
CI	confidence interval
CIMP	CpG island methylator phenotype
*COX-2*	cyclooxygenase-2
CRC	colorectal cancer
*CTNNB1*	catenin beta 1
DNA	deoxyribonucleic acid
dsDNA	double-stranded DNA
EGFR	epidermal growth factor receptor
EOCRC	early-onset colorectal cancer
FFPE	formalin-fixed, paraffin-embedded
FDR	false discovery rate
FDA	Food and Drug Administration
*FGFR3*	fibroblast growth factor receptor 3
*FBXW7*	F-box and WD repeat domain containing 7
*GNAS*	GNAS complex locus (G protein subunit alpha)
H&E	hematoxylin and eosin
*HNF1A*	hepatocyte nuclear factor 1 alpha
IBD	inflammatory bowel disease
*IDH1*	isocitrate dehydrogenase 1
*KRAS*	Kirsten rat sarcoma viral oncogene homolog
LOCRC	later-onset colorectal cancer
*MAPK*	mitogen-activated protein kinase
MMR	mismatch repair
MSI-H	microsatellite instability-high
MSS	microsatellite stable
NGS	next-generation sequencing
*NOTCH1/3*	neurogenic locus notch homolog 1/3
NSAIDs	nonsteroidal anti-inflammatory drugs
NOS	not otherwise specified
*NRAS*	neuroblastoma RAS viral oncogene homolog
OR	odds ratio
*PCR*	polymerase chain reaction
*PI3K*	phosphoinositide 3-kinase
*PIK3CA*	phosphatidylinositol-4,5-bisphosphate 3-kinase catalytic subunit alpha
PM	personalized medicine
*RB1*	retinoblastoma 1
*RNF43*	ring finger protein 43
*SD*	standard deviation
SEER	Surveillance, Epidemiology, and End Results
*SMAD4*	SMAD family member 4
SPSS	Statistical Package for the Social Sciences
TMB	tumor mutational burden
TKI	tyrosine kinase inhibitor
TNM	Tumor–Node–Metastasis
*TP53*	tumor protein p53
UICC	Union for International Cancer Control
