# Supplementary material for: Age-Related Multigene Analysis of Colorectal Cancer Using Next-Generation Sequencing
Source: Cancers (Basel). 2025 Dec 6;17(24):3909. doi: 10.3390/cancers17243909 (PMC12730896; doi:10.3390/cancers17243909)
Supplement: Supplementary file 1 [file cancers-17-03909-s001.zip › cancers-3953905-supplementary.pdf]

Supplementary Table 1: Pathway Classification with Protein Changes

Legend:

RAS=KRAS, NRAS

TP53 pathway=TP53 mutations

PI3K pathway=*PIK3CA, PTEN*

WNT pathway=*APC, CTNNB1*

TGFβ=*SMAD4*

Other=*BRAF, ALK, ERBB* family, *FBXW7, GNAS, HNF1A, IDH1, ATM*

Case	Age	T_stage	RAS	TP53	PI3K	WNT	TGFβ/Other
1	36	pT3				*APC* p.S1465Wfs*2	*SMAD4* c.G400A; *IDH1* p.R132H
2	57	pT3	*KRAS* p.G12V			*APC* p.R876X	
3	57	pT1	*KRAS* p.G12D; *KRAS* p.G12V	*TP53* p.G244S		*APC* p.D1486Ifs*20	
4	55	pT3			*PIK3CA* p.A1035V	*APC* p.E1513X	*FBXW7* p.R393X; *HNF1A* p.R200W
5	51	pT3	*KRAS* p.G12D	*TP53* p.R273C			
6	47	pT2	*NRAS* p.G13R	*TP53* p.Y220C	*PIK3CA* p.G1050S	*APC* p.S1346X	
7	54	pT3	*KRAS* p.G12R				
8	31	pT3		*TP53* p.R282W	*PTEN* p.V428A		
9	44	pT3		*TP53* p.S215I			*BRAF* p.V600E
10	46	pT4a		*TP53* p.F270I			

11	44	pT2		*TP53* p.Q167_V172delinsH	*PIK3CA* p.Q546K		
12	40	pT3	*KRAS* p.G12V	*TP53* p.R213X	*PIK3CA* p.H1047Y	*APC* p.E1309X	
13	50	pT3	*NRAS* p.G12D			*APC* p.S1356X	*BRAF* p.G466R
14	39	pT2	*KRAS* p.G13D			*APC* p.Q1096X	
15	54	pT1	*KRAS* p.G12D			*APC* p.R876X	
16	53	T4b	*KRAS* p.G12D	*TP53* p.R273C			*SMAD4* p.A118E
17	53	pT4b				*APC* p.E1353Dfs*20	*FBXW7* p.R278X
18	56	pT4a		*TP53* p.R175H		*APC* p.E1309Dfs*3	*ERBB4* p.R938H
19	52	pT3	*KRAS* p.G12A	*TP53* p.R213X		*APC* p.E1309Dfs*3	
20	43	pT4a		*TP53* p.Q165Hfs*4			
21	49	pT3	*KRAS* p.G12V	*TP53* p.R196X		*APC* p.E1577Dfs*72	
22	57	pT3	*KRAS* p.G13D	*TP53* p.R213X	*PIK3CA* p.N345H; *PIK3CA* p.R108H; *PIK3CA* p.R88Q; *PTEN* p.R406Q	*CTNNB1* p.S38F	*SMAD4* p.D120Y
23	53	pT4a		*TP53* p.R175H			*BRAF* p.V600E
24	57	pT3	*KRAS* p.G12V	*TP53* p.N210Kfs*5			
25	56	pT2	*KRAS* p.G12V	*TP53* p.R273H		*APC* p.S1321Rfs*9	
26	56	pT2	*KRAS* p.G12S			*APC* p.S1346X	
27	46	pT4a	*KRAS* p.G13C	*TP53* p.S94X			
28	56	pT4a		*TP53* p.G245V			
29	43	pT1	*NRAS* p.G13R			*APC* p.R876X	

30	44	pT4a		*TP53* p.K305Sfs*39			
31	48	pT3	*KRAS* p.G12A	*TP53* p.G266R		*APC* p.F1491Lfs*15	*SMAD4* p.A406T; *FBXW7* p.R465L; *GNAS* p.R201H
32	36	pT4a	*KRAS* p.G12C				*ATM* p.R3008C
33	45	pT4a	*NRAS* p.Q61K	*TP53* p.R342X			
34	40	pT3	*NRAS* p.G12D	*TP53* p.K132T		*APC* p.E1309Dfs*3	
35	49	pT4	*NRAS* p.G12D			*APC* p.E1309Dfs*3	
36	50	pT3	*KRAS* p.A146T	*TP53* p.L257P		*APC* p.Q1291X	
37	70	pT4a	*KRAS* p.G12D		*PIK3CA* p.E545K		
39	70	pT1	*KRAS* p.G12V				
40	65	pT3	*KRAS* p.G12D		*PIK3CA* p.M1040K; *PTEN* p.R346H		
41	92	pT4b		*TP53* p.R267W; *TP53* p.Y103X			
42	67	pT3	*KRAS* p.G12D	*TP53* p.R175H			
43	86	pT4a	*KRAS* p.A146T	*TP53* p.R175H	*PIK3CA* p.H1047R		*FBXW7* p.R465H
44	74	pT2	*KRAS* p.G13D	*TP53* p.R181H		*APC* p.S1495Kfs*12	*SMAD4* p.R135X
45	42	pT3	*KRAS* p.G12A	*TP53* p.R282W		*APC* p.E1309X	
46	67	pT3	*KRAS* p.G12V		*PIK3CA* p.Y1021C	*APC* p.R876X	
47	88	pT3	*KRAS* p.K117N		*PIK3CA* p.E542K		
48	69	pT3	*KRAS* p.G13D			*CTNNB1* p.T34A	*ALK* p.R1181H
49	57	pT2		*TP53* p.Y220C			*IDH1*

							p.N116Kfs*27
50	68	pT3		*TP53* p.M133K		*APC* p.Q1367X	
51	55	pT3	*KRAS* p.G12A	*TP53* p.R248W		*APC* p.E1309Dfs*3	*FBXW7* p.R473Kfs*3
52	56	pT2	*KRAS* p.G12V			*APC* p.R876X	*FBXW7* p.S582L
53	76	pT3	*KRAS* p.G12S	*TP53* p.V173L		*APC* p.K1370Vfs*43	
54	66	pT3		*TP53* p.V73Wfs*49			*BRAF* p.V600E
55	58	pT3	*KRAS* p.G12V		*PIK3CA* p.E542K; *PIK3CA* p.N345I		*SMAD4* p.A460Gfs*42; *SMAD4* p.R445X
